# Supplementary material for: Chronic Alcohol Exposure Promotes Cancer Stemness and Glycolysis in Oral/Oropharyngeal Squamous Cell Carcinoma Cell Lines by Activating NFAT Signaling
Source: Int J Mol Sci. 2022 Aug 29;23(17):9779. doi: 10.3390/ijms23179779 (PMC9456298; doi:10.3390/ijms23179779)

Suppl. Figure S1

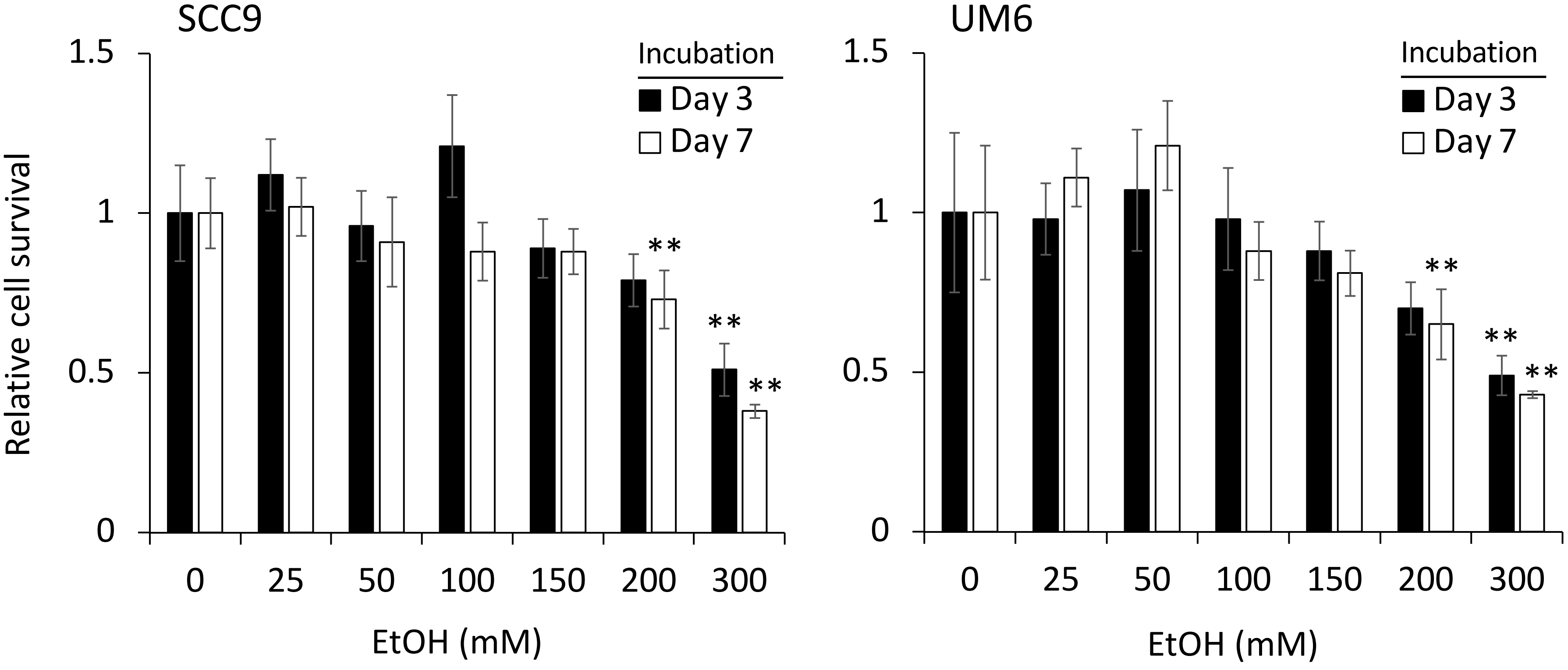

Suppl. Figure S2

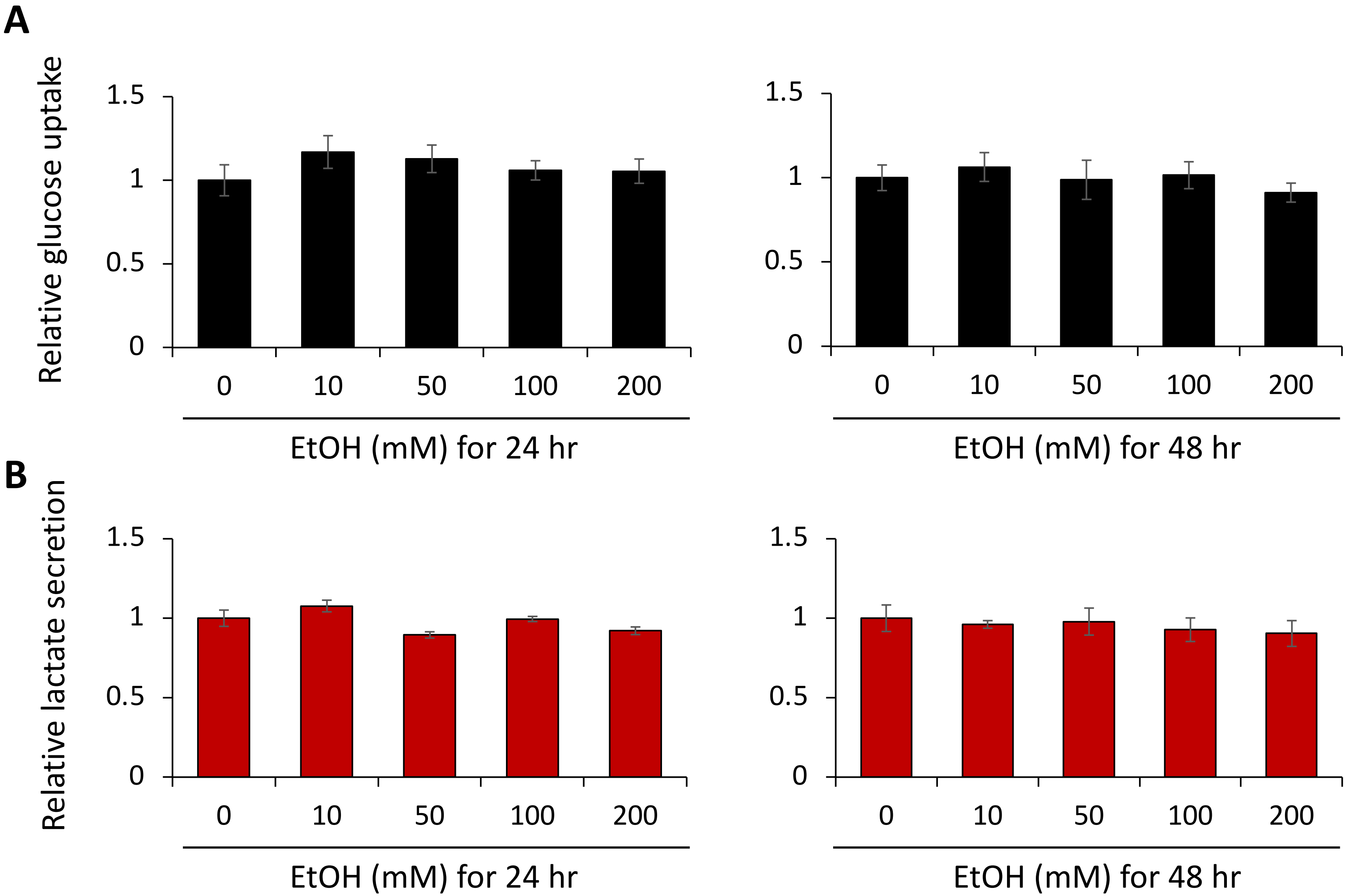

Suppl. Figure S3

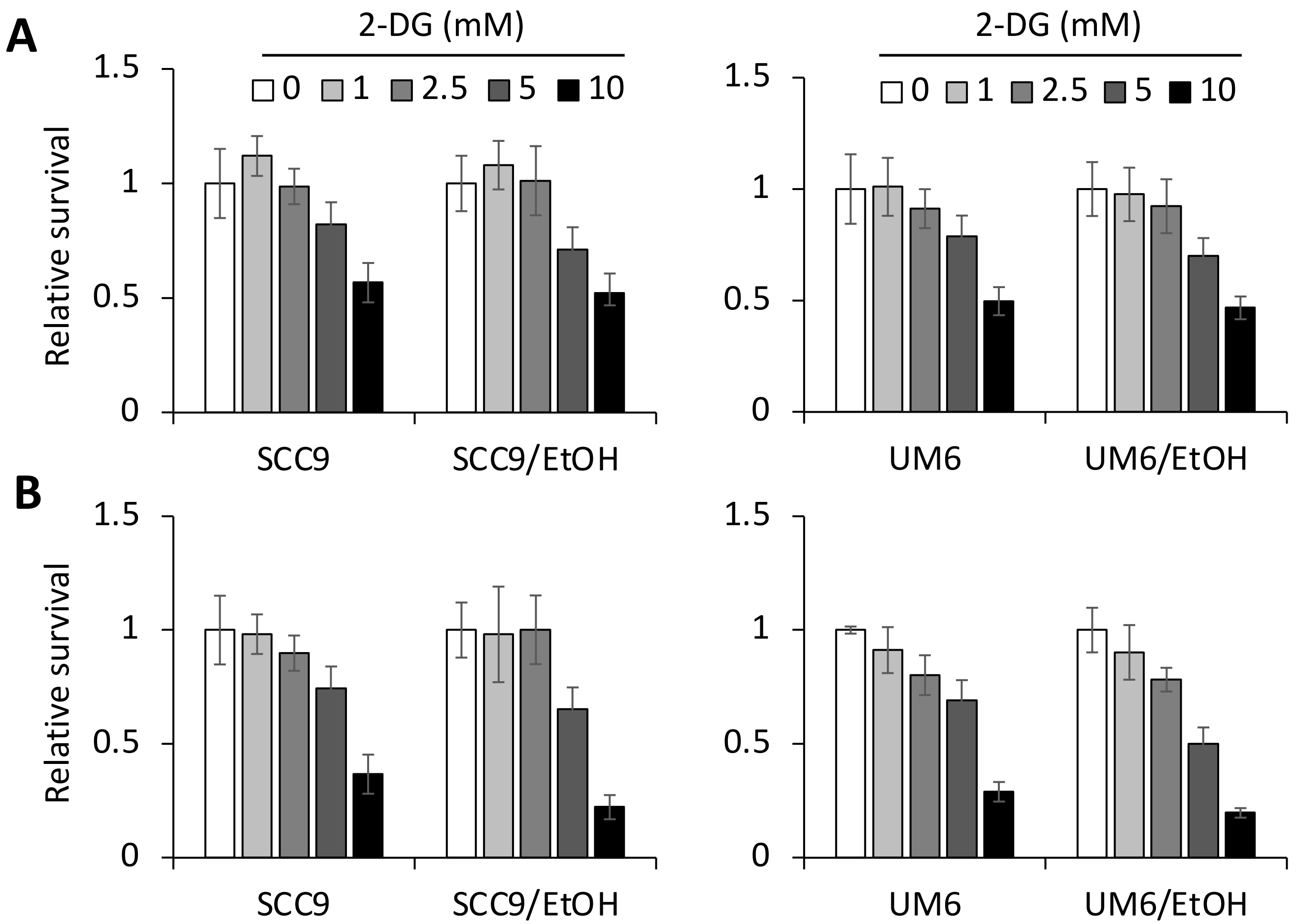

Suppl. Figure S4

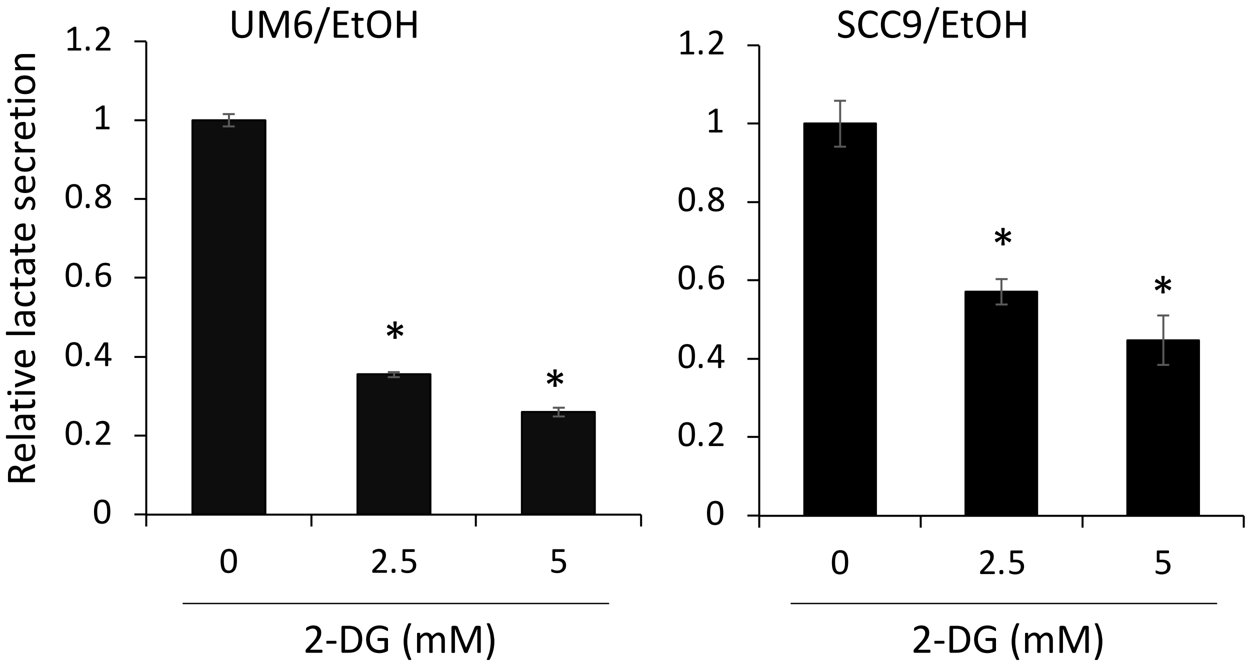

Suppl. Figure S5

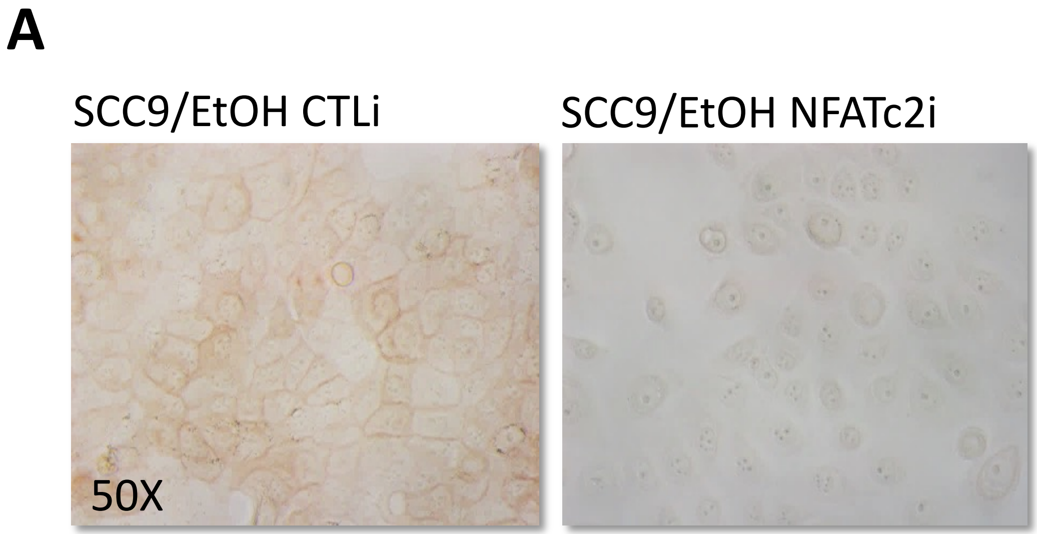

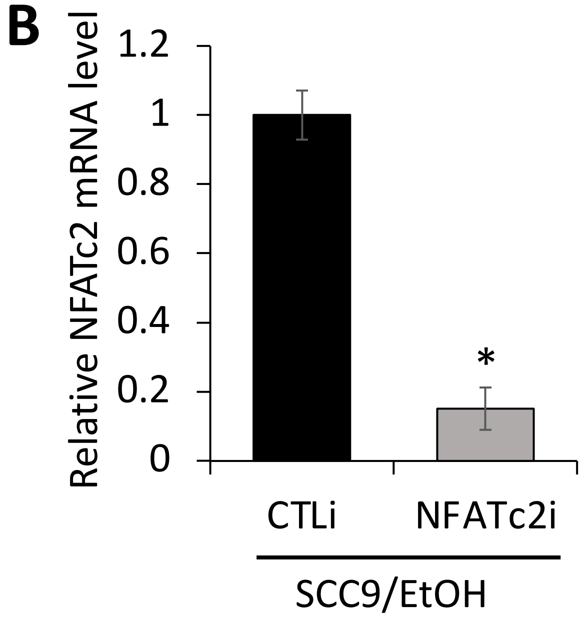

Suppl. Figure S6

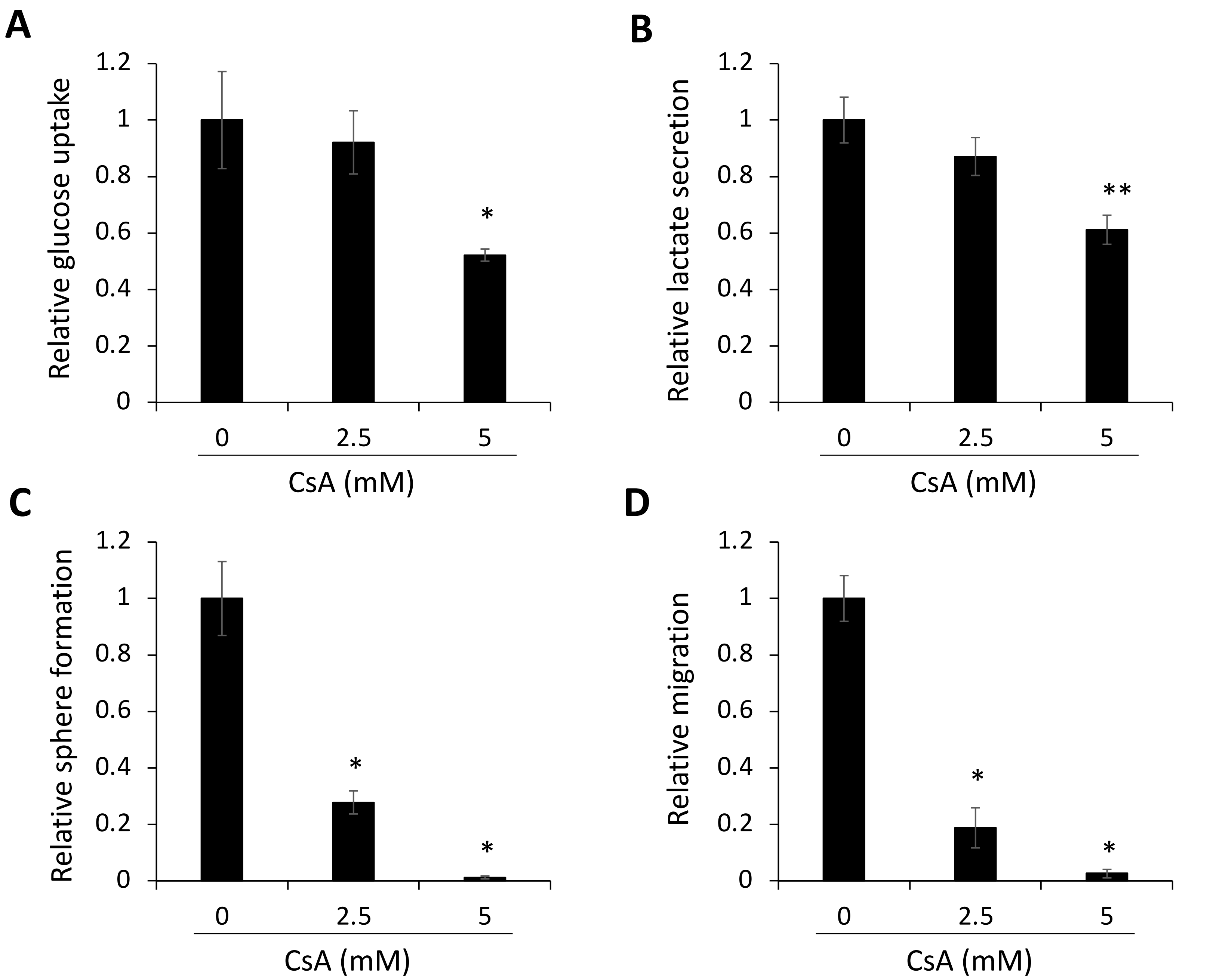

Supplement: Supplementary file 1 [file ijms-23-09779-s001.zip › ijms-1803455-supplementary.pdf]
